# Decentralized 3D Collision Avoidance for Multiple UAVs in Outdoor Environments

**DOI:** 10.3390/s18124101

**Published:** 2018-11-23

**Authors:** Eduardo Ferrera, Alfonso Alcántara, Jesús Capitán, Angel R. Castaño, Pedro J. Marrón, Aníbal Ollero

**Affiliations:** 1 Networked Embedded Systems Group, University of Duisburg-Essen, 45127 Essen, Germany; pjmarron@uni-due.de; 2 Group of Robotics, Vision and Control, University of Seville, 41092 Seville, Spain; aamarin@us.es (A.A.); jcapitan@us.es (J.C.); castano@us.es (A.R.C.); aollero@us.es (A.O.)

**Keywords:** multi-UAV, collision avoidance, decentralized coordination

## Abstract

The use of multiple aerial vehicles for autonomous missions is turning into commonplace. In many of these applications, the Unmanned Aerial Vehicles (UAVs) have to cooperate and navigate in a shared airspace, becoming 3D collision avoidance a relevant issue. Outdoor scenarios impose additional challenges: (i) accurate positioning systems are costly; (ii) communication can be unreliable or delayed; and (iii) external conditions like wind gusts affect UAVs’ maneuverability. In this paper, we present 3D-SWAP, a decentralized algorithm for 3D collision avoidance with multiple UAVs. 3D-SWAP operates reactively without high computational requirements and allows UAVs to integrate measurements from their local sensors with positions of other teammates within communication range. We tested 3D-SWAP with our team of custom-designed UAVs. First, we used a Software-In-The-Loop simulator for system integration and evaluation. Second, we run field experiments with up to three UAVs in an outdoor scenario with uncontrolled conditions (i.e., noisy positioning systems, wind gusts, etc). We report our results and our procedures for this field experimentation.

## 1. Introduction

The use of multiple Unmanned Aerial Vehicles (UAVs) that cooperate to perform some tasks is becoming mainstream, mostly due to the flexibility and breadth of their mobility and sensing capabilities as well as the advancement of associated technologies. For instance, teams of UAVs are being used in applications related to transportation [1], delivery of goods [2] or even cinematography [3]. Many works address the problem of avoiding collisions while navigating such teams in indoor facilities. However, outdoor scenarios bring extra difficulties since positioning systems are not always accurate, communication can be unreliable, weather conditions can make maneuverability more complex, etc.

Since applications requiring UAVs that operate jointly in a shared airspace are spreading, methods that cope with 3D collision avoidance under the above constraints are of uppermost importance. This problem is commonly tackled in the literature in a centralized fashion, but outdoor scenarios demand robustness against faulty communication systems and scalability. Therefore, we focus on decentralized approaches that can scale better with the number of vehicles and size of the scenario. Moreover, we prioritize safety and fast responses in our system over optimality of the trajectories. We aim at a reactive approach where UAVs do not optimize their trajectories for a time horizon ahead, but instead ensure their safety under harsh sensor and communication constraints with a myopic algorithm not computationally expensive.

We propose a decentralized algorithm to solve the problem of 3D collision avoidance with an outdoor fleet of UAVs. We eliminate the need for a central entity with access to all the information, and each UAV uses only local measurements and communication. The algorithm operates reactively with low computational overhead and allows UAVs to integrate measurements from their local sensors as well as positions sent by teammates within communication range. The main idea is that each UAV creates a reserved space around itself and detects hypothetical future collisions (i.e., conflicts) when there are obstacles entering that space. If conflicts are detected, the UAVs maneuver avoiding each other without colliding. It is assumed that all UAVs follow the same rules in order to converge to a solution. In particular, we extend our previous work in 2D collision avoidance [4], where we developed the algorithm SWAP (Safety-enhanced avoidance policy), and present in this paper a 3D version for UAVs called 3D-SWAP.

We contribute in this paper in two main aspects:

- First, we propose 3D-SWAP, a novel algorithm for 3D collision avoidance with multiple UAVs. The algorithm extends ideas from our previous work on ground robots that swap their positions in a traffic roundabout fashion. Here, a similar strategy on a horizontal plane is combined with a control of the UAVs’ altitude to navigate safely in 3D environments. Thus, UAVs that are far enough in altitude can ignore each other, making the swapping of the rest more efficient. Moreover, our approach requires low computational load, is decentralized and works with noisy sensors and restricted communication.
- Second, we detail our system architecture and the implementation of our method in a real team of UAVs. We tested our algorithm in realistic simulations to assess its performance. Later, we also run tests in outdoor field experiments, coping with noisy communication, inaccurate positioning systems, wind gusts, etc. We explain our procedures for the development and integration of the algorithm in these field experiments.

The remainder of this paper is organized as follows: Section 2 discusses related work; Section 3 formulates the problem; Section 4 provides all the details about our algorithm 3D-SWAP; Section 5 analyzes further 3D-SWAP; Section 6 describes the system integration and experimental results; and Section 7 includes conclusions and future work.

## 2. Related Work

The problem of motion planning is a classical problem in robotics. Traditionally, it has been divided into global path planning and local path planning, also called collision avoidance. In the former, a robot must find a path free of collision from an initial to a final state in an environment that may contain static and/or dynamic obstacles. In the latter, the objective is to compute collision-free paths for a shorter time horizon, i.e., to navigate safely reacting to obstacles that were not planned in advance. If multiple autonomous vehicles are considered in a 3D scenario, the problem is harder. This is the case in this paper, where we address collision avoidance in 3D environments for multi-UAV settings. A complete review for UAV motion planning can be seen in [5].

In a multi-vehicle scenario, global path planning is often formulated as a constrained optimization problem. Centralized approaches are usual for optimal trajectory generation. In [6], the problem is formulated as a Mixed Integer Program, which works in continuous space and takes dynamics into account to compute trajectories for quadrotors. Another approach is used in [7], where optimal multi-robot path planning is solved by means of discrete graphs. There are some issues with methods based on centralized optimization. Although they provide optimal solutions, they are usually computationally expensive. Besides, solvers become more complex for non-convex problems, which is the case quite often in multi-robot collision avoidance. Due to this, we favor safety instead of optimality in 3D-SWAP. Nonetheless, there are still some efficient solutions for non-convex problems achieving local optimality. For instance, in non-convex settings, Sequential Convex Programming is a technique to find feasible solutions by means of convex approximations. In [8], the method is used with a real-time implementation for a team of quadrotors.

Constrained optimization algorithms have also been proposed for local collision avoidance in multi-UAV teams. For example, Turpin et al. propose a centralized approach where the problem is modeled as a task allocation [9]. Then, optimal trajectories in terms of traveled distance are generated for multiple UAVs. The same authors also presented a decentralized version of the algorithm [10], where UAVs use only local observations and communication. Moreover, methods based on formation control can also be used to navigate multiple UAVs safely to their goals. The objective is to solve an optimal control problem to maintain desired formations, e.g., by imposing inter-robot distances or angles. A complete review can be found in [11]. Formation control can be solved in a centralized [6,12] or decentralized fashion [13]. In [12], centralized linear programming is proposed in a velocity space for multi-robot formation. In [13], sequential convex programming is applied to distributed optimization through a consensus-based algorithm. In general, formation control is interesting for coupled teams of vehicles. These appear in applications that imply a group of robots navigating together to perform a common task, such as tracking an object or transporting something jointly. However, formation-based methods reduce their applicability in other scenarios where vehicles need to navigate to goal positions independently.

In this work, we aim at decentralized approaches, where each vehicle can only access local observations (i.e., from onboard sensors) and use local communications (i.e., with its neighbors). These approaches present advantages in outdoor scenarios, mainly for large-scale applications. They can scale better with the number of UAVs and they do not rely on a central communication facility, which is not usually available in the mentioned scenarios. Many works in decentralized collision avoidance rely on the concept of Velocity Obstacles (VO), where collision-free trajectories are computed by imposing constraints in the velocity space. For instance, ref. [14] propose a convex optimization method to solve local collision avoidance based on Velocity Obstacles. They solved the problem in a centralized manner, but then present a decentralized solution which considers multiple UAVs and static obstacles. A similar decentralized optimization method is proposed in [15] using Model Predictive Control, but uncertainties in states’ estimation are also considered. Lalish et al. also present a decentralized algorithm to apply Velocity Obstacles to 3D collision avoidance [16]. They analyze the robustness of the algorithm against unmodeled dynamics and non-cooperative vehicles.

A disadvantage of methods based on Velocity Obstacles is that they rely on an accurate estimation of the velocity of surrounding obstacles, which is not always straightforward. Although some works consider uncertainties in the position estimations [15] or unmodeled dynamics [16], they assume that UAVs can observe velocities from others, something that we avoid in our solution. Moreover, 3D-SWAP is proved to behave safely against localization uncertainties. This links with another limitation of the applicability of many of the works mentioned so far, which is that they are only validated through simulations or make use of a Vicon system [15]. Outdoor settings cannot rely on fixed, high-precision positioning systems such as a Vicon. Instead we consider more common localization systems like usual GPS receivers (i.e., no RTK GPS), which are noisier but have lower prices.

Another option for outdoor collision avoidance with UAVs are methods based on potential fields or bio-inspired. In [17], an outdoor GPS and vision-based swarm with ten UAVs is presented. Potential fields are used for collision avoidance in a bio-inspired and decentralized flocking approach. Price et al. propose an algorithm for target cooperative tracking with multiple UAVs outdoors [18]. They use potential fields for collision avoidance in order to circumvent the non-convexity of their Model Predictive Controller for formation control. Moreover, artificial potential fields are use in [19] for local obstacle avoidance given a 3D occupancy grid; and in [20], a heuristic-driven 3D visibility graph is proposed for local navigation. These two works focus on the complete architecture for localization, mapping and navigation for a single UAV, providing a thorough insight into implementation details, but do not concentrate on the multi-vehicle problem. In general, methods based on potential fields can be easily decentralized and imply low computational requirements. However, they can present issues in terms of convergence and local minima when many vehicles concentrate on the same area. Our proposal of ignoring vehicles far enough in altitude and swapping positions orderly on a horizontal plane for the rest turns out to be efficient for those situations.

Finally, there is also extensive literature regarding see-and-avoid methods, which aim to integrate unmanned aircrafts into the civil airspace. A recent and complete review of these methods can be found in [21]. They follow current regulation in terms of trajectories and use typically visual sensors for obstacle detection. For instance, in [22] a visual predictive control approach is proposed following spiral trajectories for collision avoidance. Nonetheless, the validation is performed by means of quadrotors with a Vicon motion capture system.

## 3. Problem Description

We assume that there is a team of UAVs operating in a 3D environment that need to navigate to their destinations without colliding. Those destinations represent local goal waypoints that may come from a higher-level motion path planner. Our problem is to navigate the UAVs safely to their local goals reacting to possible collisions with other UAVs and with external obstacles. By external obstacles we imply existing static obstacles in the scenario different from the UAVs in the team. Formally, given a set of *N* UAVs, let ${\left\{p_{i}\left(t\right)=\left(p^{xy},z\right)|p_{i}\left(t\right)\in R^{3}\right\}}_{i=1,\ldots ,N}$ be their 3D positions at each time instant *t*, where $p^{xy}$ is the 2D projection onto the xy-horizontal plane and *z* is the altitude. Also, let ${\left\{g_{i}|g_{i}\in R^{3}\right\}}_{i=1,\ldots ,N}$ be their goal waypoints.

First, let us define the *UAV coordinate frame*, which is a coordinate frame associated with each UAV *i* and centered at its position $p_{i}$. The x-axis is aligned with the UAV yaw orientation and the z-axis is vertical, pointing upwards. The xy-plane of this UAV coordinate system stays always horizontal and does not tilt or roll with UAV movements. Then, we can define the concept of collision.

**Definition** **1.** ***(Collision)** We define the collision hull of each UAV as a cylinder that circumvents its shape. The collision cylinder has radius $r_{c}$ and height $h_{c}$, and its center coincides with the UAV position. The vertical axis of this cylinder is aligned with the UAV z-axis. A collision occurs when the collision cylinders of two UAVs overlap or when an external obstacle enters the collision cylinder of a UAV.*

Therefore, if we denote $C_{i}$ as the collision cylinder of UAV *i* and $V_{k}$ as a 3D volume representing external obstacle *k*, a collision for UAV *i* occurs if ∃j≠i such that $C_{i}\cap C_{j}\neq \emptyset$ or ∃k such that $C_{i}\cap V_{k}\neq \emptyset$. Our objective is to navigate each UAV *i* to its goal $g_{i}$ safely, i.e., with no collision at any moment.

It is important to note that modeling the shape of the UAVs as cylinders will be helpful for checking collisions efficiently and, at the same time, it makes sense for multirotors due to the downwash effect on other vehicles [9,14] (the safety separation in vertical distance should be larger than in horizontal to avoid perturbations). Moreover, to be strict, the collision cylinder should tilt and roll as the UAV does. Instead, we assume it fixed to the UAV coordinate frame, which is always horizontal. This simplifies our solution and makes sense provided that the collision cylinder is big enough to encompass the UAV shape even tilted or rolled.

Additionally, we make the next assumptions in our problem:

- *Holonomic vehicles*. We model UAVs as holonomic vehicles (e.g., multirotors). They can move in any direction independently from their yaw orientation. We assume that their acceleration and speed constraints allow them to stop horizontally within a planar breaking distance $d_{br}$, and stop their vertical movement within a vertical distance $z_{br}$.
- *Noisy localization*. UAVs can localize themselves by means of noisy sensors. In outdoor scenarios, UAVs could carry GPS receivers and altimeters, for instance. Each UAV has access to its own noisy localization $p^{\prime }=\left({p^{\prime }}^{xy},z^{\prime }\right)$, such that $||{p^{\prime }}^{xy}-p^{xy}||\leq \epsilon_{xy}$ and $|z^{\prime }-z|\leq \epsilon_{z}$. $\epsilon_{xy}$ and $\epsilon_{z}$ are the maximum localization errors on the xy-plane and altitude, respectively. We differentiate them, as altitude is usually more precise due to the use of altimeters or lasers.
- *Local communication*. If two UAVs *i* and *j* are within communication range, i.e., $||p_{i}-p_{j}||\leq r_{comm}$, they can exchange their noisy localizations $p_{i}^{\prime }$ and $p_{j}^{\prime }$. Thus, UAVs share with their neighbors their position, but not their goals, velocities nor orientations. We do not assume a perfect communication. When communication links fail, other UAVs could still be detected with the onboard sensors.
- *Obstacle detection*. UAVs have onboard sensors to detect obstacles within a 3D distance $r_{det}$. In particular, we assume that each UAV has a 3D sensor generating poincloud-based measurements from the obstacles around (e.g., a Lidar). Each pointcloud consists of *M* points, where each point *m*, without losing generality, can be expressed in cylindrical coordinates relative to the UAV $\left(\rho_{m},\varphi_{m},z_{m}\right)$. Again, we assume those measurements to be noisy.

In our problem formulation, the 3D sensors onboard are primarily used to detect external obstacles. In order to detect other UAVs, the communication channel can be used. However, we do not assume perfect communication, as it could fail even if UAVs are within communication range. Therefore, the 3D sensors could also be used to detect other teammates when there are communication losses. Of course, we rely on the fact that all external obstacles or teammates will be detected on time one way or another.

## 4. 3D-SWAP

In this section we describe 3D-SWAP, which is a novel algorithm to solve the collision avoidance problem formulated in Section 3 in a decentralized fashion. In particular, we develop an extension for aerial vehicles of our previous algorithm SWAP [4], which addressed collision avoidance for large teams of ground robots.

### 4.1. Overview and Preliminaries

**Figure 1:**
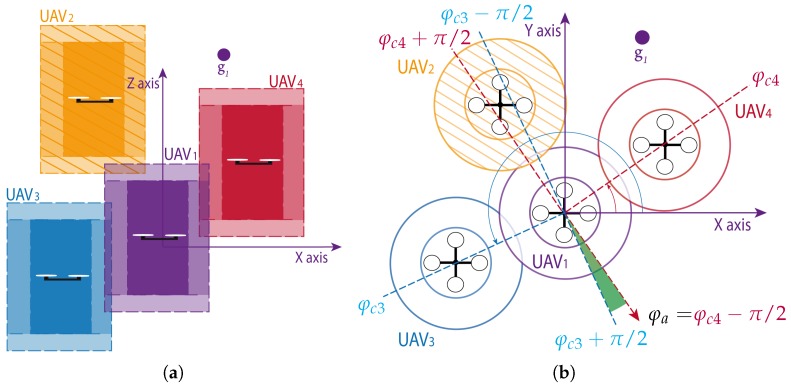
Example situation with several UAVs in conflict. The figure shows the avoidance maneuver computed by 3D-SWAP for UAV 1 while trying to reach its goal $g_{1}$. All UAVs are represented with their collision, reserved and blocking cylinders. On the **left** (**a**), a lateral view of the scene to depict z-conflict with UAV 2. On the **right** (**b**), a view from above to show xy-conflicts with UAV 3 and 4, and the computation of the avoidance direction.

The original SWAP [4] is a reactive algorithm for collision avoidance in ground multi-robot teams. Safety disks are defined around each robot to detect possible conflicts when obstacles enter these safety areas. These conflicts are then resolved in a decentralized fashion by means of a set of maneuvers where all robots follow similar predefined rules. 3D-SWAP generalizes SWAP to deal with UAVs in 3D spaces. First, the notion of disks is extended to cylinders. By defining different cylinders around each UAV and checking when they are invaded, vehicles can detect two different types of conflicts: *xy-conflicts* when others get too close on an xy-horizontal plane; and *z-conflicts* when others get too close in altitude. If an xy-conflict is detected, the vehicles involved move surrounding each other laterally in a roundabout fashion, i.e., swapping their positions at the same time that they keep going up or down in altitude as they were. If a z-conflict is detected, the vehicles involved keep moving as toward their goals, but fixing their altitude while the z-conflict persists, not to get closer vertically. Figure 1 depicts an example where a UAV 1 is surrounded by several others to understand the two types of conflicts that can occur in 3D-SWAP and how they are resolved (a video illustrating this example and another one with more UAVs can be found at https://youtu.be/-iiPJ9vuUA8). UAV 1 has a z-conflict with UAV 2 (see Figure 1a), so it needs to keep its altitude fixed instead of flying up toward its goal. This is to prevent a possible collision with UAV 2. Besides, UAV 1 has xy-conflicts with UAVs 3 and 4, which are close on the same horizontal plane. UAV 1 will compute an avoidance direction $\varphi_{a}$ in order to move horizontally surrounding UAV 4 counter-clockwise toward its goal (see Figure 1b).

**Figure 2:**
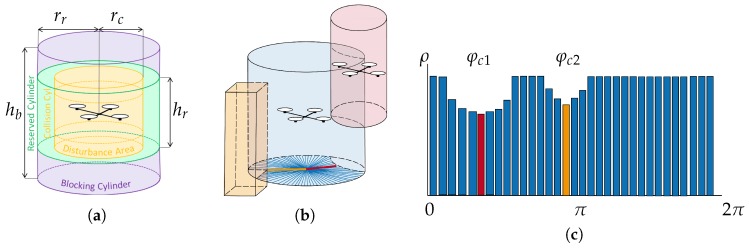
(**a**) Representation of the three concentric cylinders that define the collisions and conflicts. (**b**) A UAV (in the middle) computes its COD with two obstacles: an external, static obstacle and another UAV. The reserved cylinder of the central UAV is shown to see how it is invaded by the collision cylinder of the other UAV and the static obstacle. (**c**) Equivalent polar representation of its COD with the two conflict angles.

More in detail, 3D-SWAP works by defining two concentric cylinders around each UAV: the *reserved cylinder* and the *blocking cylinder* (see Figure 2a). These cylinders are the foundation to define the concept of conflict and navigate UAVs without collision to their destination.

**Definition** **2.** ***(Reserved cylinder)** The reserved cylinder has radius $r_{r}$ and height $h_{r}$ and its volume is denoted as R. It is concentric and aligned with the collision cylinder. This cylinder is used to detect conflicts on the xy-plane. In particular, a UAV i has an xy-conflict when its reserved cylinder intersects with another reserved cylinder or with an external obstacle. This means that ∃j≠i such that $R_{i}\cap R_{j}\neq \emptyset$ or ∃k such that $R_{i}\cap V_{k}\neq \emptyset$. The radii $r_{r}$ must ensure that UAVs can always detect xy-conflicts on time to brake horizontally before a lateral collision, and then maneuver accordingly.*

**Definition** **3.** ***(Blocking cylinder)** The blocking cylinder is concentric and aligned with the reserved cylinder, having the same radius but height $h_{b}$, and its volume is denoted as B. It is defined by adding to the reserved cylinder a cylinder $B^{+}$ on top and another $B^{-}$ below, such that $B=B^{+}\cup R\cup B^{-}$. The blocking cylinder is used to detect conflicts in altitude. Thus, a UAV i has a z-conflict when the $B^{+}$ or $B^{-}$ part of its blocking cylinder intersects with the $B^{-}$ or $B^{+}$ (respectively) part of another blocking cylinder or with an external obstacle. This means that: (i) ∃j≠i such that $B_{i}^{+}\cap B_{j}^{-}\neq \emptyset$ or $B_{i}^{-}\cap B_{j}^{+}\neq \emptyset$; or (ii) ∃k such that $B_{i}^{+}\cap V_{k}\neq \emptyset$ or $B_{i}^{-}\cap V_{k}\neq \emptyset$. The height $h_{b}$ must ensure that UAVs can always detect z-conflicts on time to brake vertically before a collision from above or below, and then maneuver accordingly.*

The complete procedure to run 3D-SWAP in a decentralized manner is depicted in Algorithm 1. An instance of this algorithm runs on each UAV in order to navigate the whole team safely toward their goals. Each UAV *i* uses its local information, i.e., its own noisy position $p_{i}^{\prime }$ and its last pointcloud observed. At each iteration, the UAV shares its position with the neighbors (Line 2) and initializes its Cylindrical Obstacle Diagram (COD) without obstacles (Line 3). Then, the COD is updated with the positions received from other neighboring UAVs (Lines 4–8) and with the information in the local pointcloud (Lines 9–12). This procedure will be detailed in Section 4.2. The conflict state of the UAV is determined by means of this COD (Line 13). Depending on its current state, the UAV performs different maneuvers varying the reference velocity to its velocity controller (Lines 15–28). These maneuvers and how the UAV movement is controlled are explained in Section 4.3.

**Algorithm 1** 3D-SWAP for each UAV *i***Input:** Pointcloud from local sensors, current position $p_{i}^{\prime }$, goal position $g_{i}$
   1:**while**$g_{i}$ not reached **do**   2: Send position $p_{i}^{\prime }$ to neighbors   3: COD←initCOD()   4: **for all** UAV *j* within communication range **do**   5:  Receive its position $p_{j}^{\prime }$   6:  $\left(\rho_{j}^{\prime },\varphi_{j}^{\prime },z_{j}^{\prime }\right)\leftarrow transformToCylindrical\left(p_{j}^{\prime }\right)$   7:  $COD\leftarrow UpdateCOD(\rho_{j}^{\prime },\varphi_{j}^{\prime },z_{j}^{\prime })$   8: **end for**   9: **for** m=1 m=M **do**  10:  Extract point *m* from local pointcloud  11:  $COD\leftarrow UpdateCOD(\rho_{m},\varphi_{m},z_{m})$  12: **end for**  13: $\left(s_{xy},s_{z}\right)\leftarrow computeState\left(COD\right)$  14: $d^{g}=g_{i}-p_{i}^{\prime }$  15: **if** $s_{xy}$ is xy-free **then**  16:  $∠v_{xy}^{ref}=∠d_{xy}^{g}$ and $\left||v_{xy}^{ref}||=computeRefSpeed(||d_{xy}^{g}||,v_{max}\right)$  17: **else if** $s_{xy}$ is rendezvous **then**  18:  $∠v_{xy}^{ref}=\varphi_{a}$ and $||v_{xy}^{ref}||=v_{a}$  19: **else if** $s_{xy}$ is xy-blocked **then**  20:  $v_{xy}^{ref}=0$  21: **end if**  22: **if** $s_{z}$ is z-free **then**  23:  $v_{z}^{ref}=\pm computeRefSpeed\left(d_{z}^{g},v_{max}\right)$, depending on whether $g_{i}$ is above or below  24: **else if** $s_{z}$ is z-blocked **then**  25:  $v_{z}^{ref}=0$  26: **end if**  27: $v^{ref}\leftarrow \left(v_{xy}^{ref},v_{z}^{ref}\right)$  28: Send $v^{ref}$ to velocity controller  29:**end while**

### 4.2. The Cylindrical Obstacle Diagram

Each UAV maintains a local *Cylindrical Obstacle Diagram* (COD) to analyze its conflicts and determine its movements accordingly. The Cylindrical Obstacle Diagram is a cylindrical representation of the obstacles around a UAV that is expressed on its coordinate frame. In order to create the COD, the UAV polar angle φ is discretized into $N_{\varphi }$ values, obtaining a set of angle bins. The COD stores the polar distance ρ corresponding to the closest obstacle within each bin. Formally, the COD of a UAV consists of a set of angle bins $\left\{\varphi_{1},\cdots ,\varphi_{N_{\varphi }}\right\}$ with their corresponding polar distances to obstacles $\left\{\rho_{1},\cdots ,\rho_{N_{\varphi }}\right\}$.

The COD is used to evaluate the conflict state of a UAV. Measurements in the COD are checked to see whether the UAV reserved cylinder is invaded by another reserved cylinder or an external obstacle, provoking an xy-conflict. The COD is updated with measurements from the local pointclouds and with positions received from other UAVs. Measurements far enough in altitude to invade the UAV reserved cylinder (i.e., not able to provoke a xy-conflict) are not included in the COD. Figure 2b,c show an example of a graphical representation of the COD for a UAV observing an external obstacle and another UAV.

First, UAVs update their COD with positions sent by other UAVs. If any other UAV *j* within communication range shares its position, its cylindrical coordinates with respect to UAV *i* are computed $\left(\rho_{j}^{\prime },\varphi_{j}^{\prime },z_{j}^{\prime }\right)$: $\rho_{j}^{\prime }$ represents the planar distance between UAVs; $\varphi_{j}^{\prime }$ is the angle between the x-axis of UAV *i* coordinate frame and UAV *j* center; and $z_{j}^{\prime }$ the difference in altitude between both. Then, a virtual circular obstacle of radius $r_{c}$ is created at the position of UAV *j* representing its collision cylinder. The COD is updated accordingly to include the border of this cylinder representing UAV *j*. The closest point corresponding to this obstacle will have distance $\rho_{j}^{\prime }=||{p^{\prime }}_{j}^{xy}-{p^{\prime }}_{i}^{xy}||-r_{c}$. Before updating the COD, it is verified whether the UAV is close enough in altitude to cause an xy-conflict . Otherwise it is discarded and not included in the COD. In particular, it is included in the COD iff $|z_{j}^{\prime }|\leq 2h_{r}/2$ (reserved cylinders overlap). All the angle bins updated with measurements from other UAVs are labeled as dynamic, since there are moving obstacles in those directions.

Second, UAVs update their COD with the *M* points of their poincloud measurement if available. Each point obstacle *m* is also transformed into cylindrical coordinates $\left(\rho_{m},\varphi_{m},z_{m}\right)$. Then, $\varphi_{m}$ determines to which bin $\varphi_{i}$ the point corresponds. If $\rho_{m}<\rho_{i}$, then $\rho_{i}=\rho_{m}$, updating the COD accordingly to keep the closest obstacle at that angle. If the obstacle point corresponds to a bin labeled as dynamic, it is assumed that it comes from another UAV, so it is treated as such As before, it is included in the COD iff $|z_{m}|\leq 2h_{r}/2-h_{c}/2$ (overlap between two reserved cylinders). If the obstacle corresponds to a bin not labeled as dynamic, it is treated as static. Therefore, it is included in the COD iff $|z_{m}|\leq h_{r}/2$ (obstacle is within the UAV reserved cylinder).

After updating the COD with all available information, xy-conflicts are double-checked. For each bin in the COD, an xy-conflict occurs when: $\rho_{i}\leq r_{r}$, for bins not labeled as dynamic (static obstacle entering the reserved cylinder); or $\rho_{i}\leq 2r_{r}-r_{c}$ for bins labeled as dynamic (reserved cylinders from 2 UAVs overlapping). Thus, sectors whose angles are in xy-conflict are marked as conflict sectors; and for each conflict sector, there is a conflict angle $\varphi_{c}$ corresponding to the closest obstacle (see Figure 2b,c. Furthermore, note that during the process of updating the COD, determining whether the UAV has also a z-conflict above or below is straightforward checking the vertical and horizontal distances with the obstacle: it is a sufficient condition to find a single obstacle point causing z-conflict.

**Figure 3:**
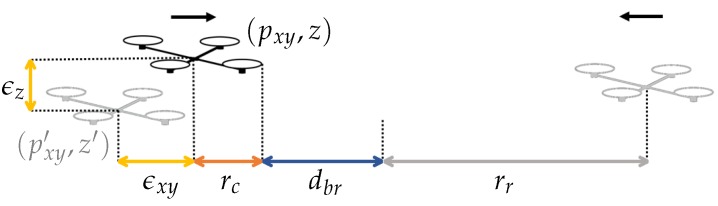
Representation of two UAVs at the instant of starting braking due to an xy-conflict. For the left UAV, the real (black) and measured (gray) positions are represented. The worst case for the maximum positioning error considered in our system is depicted.

3D-SWAP relies on the assumption that all conflictive UAVs or external obstacles are detected in time to avoid collisions. Therefore, the dimension of the cylinders must be designed in such a way that UAVs can brake safely after detecting conflicts. The reserved cylinder is used to avoid collisions given xy-conflicts, so $h_{r}=h_{c}$ and $r_{r}>r_{c}+d_{br}+\epsilon_{xy}$. Thus, once an xy-conflict is detected, UAVs would be far enough to brake in the worst case, i.e., if they were moving at full speed against each other (see Figure 3). The blocking cylinder is used to avoid collisions given z-conflicts, so $r_{b}=r_{r}$ and $h_{b}>h_{c}+z_{br}+\epsilon_{z}$. The positions communicated by the UAVs are noisy, so they may be actually closer than they think when sharing positions and computing conflicts. This is why $\epsilon_{xy}$ and $\epsilon_{z}$ are added to the braking distances in order to account for those inaccuracies on the horizontal and vertical positioning observations, respectively. We need to ensure braking in time even if measurements from the communication channels are noisy. Measurements from the pointclouds provided by the local sensors are also noisy, but we do not model them here because these inaccuracies would be typically lower than those of the positioning system, which are already included in $\epsilon_{xy}$ and $\epsilon_{z}$. Last, note that all obstacles in conflict should be detected in time to react. Therefore, we assume that $r_{comm}>2r_{r}$ (UAVs in conflict are always within communication range) and $r_{det}>r_{r}$ (any external object that enters in conflict is within detection range).

### 4.3. Avoidance Maneuvers

Each UAV computes its own COD and solves its conflicts in a decentralized fashion, performing avoidance maneuvers when needed. A UAV uses its COD to determine its conflict state. Depending on its current conflicts, the UAV can be in several states that form a state machine. Depending on the state, a 3D reference velocity is computed to control the UAV movement. UAVs assume that their neighbors will also follow the same rules to resolve their conflicts. In particular, there are two parallel state machines: one reasons about xy-conflicts and outputs a 2D reference velocity $v_{xy}^{ref}$ to move the UAV on the horizontal plane; whereas the other reasons about z-conflicts and outputs a scalar reference velocity $v_{z}^{ref}$ to move the UAV vertically. The UAV conflict state consists of a joint state from both state machines $\left(s_{xy},s_{z}\right)$, where $s_{xy}\in \left\{\mathrm{xy-free},\mathrm{redezvous},\mathrm{xy-blocked}\right\}$ and $s_{z}\in \left\{\mathrm{z-free},\mathrm{z-blocked}\right\}$. At each iteration, 3D-SWAP revises the conflict states, and both state machines can transition freely and independently if the UAV circumstances changed. Any time the UAV transitions to another state, its reference velocities change too, modifying its behavior. These reference velocities are combined into a 3D reference velocity $v^{ref}$ which is sent to a velocity controller. The design of this controller is out of the scope of the paper and we assume that the UAV autopilot provides such a functionality. Next, we describe all possible conflict states for the UAVs and their corresponding reference velocities.

**xy-free**: This is the normal operation mode for the horizontal movement. A UAV is in this state when there are no xy-conflicts, or the existing ones do not interfere with its path to its goal. The UAV should move horizontally toward its goal to reach it. For that, a 3D direction vector $d^{g}$ from the UAV to its goal is computed (Line 14, Algorithm 1). Then, the 2D reference velocity points to the goal $∠v_{xy}^{ref}=∠d_{xy}^{g}$, being $d_{xy}^{g}$ the projection of $d^{g}$ on a horizontal plane. The reference speed is computed proportional to the distance to goal and bounded by the maximum allowed speed $v_{max}$ (Line 16, Algorithm 1).

If a UAV finds xy-conflicts affecting the path to its goal, i.e. there may be a collision navigating straight to the goal, its horizontal movement is constrained to avoid the collision through two states: rendezvous and xy-blocked.

**rendezvous**: The UAV is in this state when it finds xy-conflicts but it can find an avoidance direction. Given the conflict angles $\varphi_{c}$ of the xy-conflicts, this avoidance direction $\varphi_{a}$ is computed. For each xy-conflict, the interval ($\varphi_{c}-\pi /2$, $\varphi_{c}+\pi /2$) define the forbidden angles. If the UAV keeps moving in any of those directions there is a risk of collision with the conflicting obstacle, otherwise not. Therefore, each obstacle can be surrounded following any of the directions that bound the forbidden sector, $\varphi_{c}-\pi /2$ or $\varphi_{c}+\pi /2$. In particular, the rule is that all UAVs should surround obstacles counter-clockwise, so the option $\varphi_{c}-\pi /2$ is used. However, that angle could be within the forbidden sector associated with another conflict, making impossible its selection. All conflicts are checked to find an avoidance direction $\varphi_{a}=\varphi_{c}-\pi /2$ out of forbidden sectors. If there is none, there is no escape direction and the UAV is blocked. An example is depicted in Figure 1b, where UAV 1 has two xy-conflicts with UAV 3 and UAV 4. UAV 2 is too far in altitude to cause a xy-conflict and hence not included in the COD and ignored to compute the avoidance direction. In green, the angles out of any forbidden sector are shown. In this case, $\varphi_{c3}-\pi /2$ is within a forbidden sector, so $\varphi_{a}=\varphi_{c4}-\pi /2$ is selected to surround UAV 4 counter-clockwise.

During the avoidance maneuver the speed of the UAV is reduced for security to a value $v_{a}\leq v_{max}$. Therefore, $∠v_{xy}^{ref}=\varphi_{a}$ and $\left|\right|v_{xy}^{ref}\left|\right|=v_{a}$.

**xy-blocked**: If no possible avoidance direction for the UAV is found following the previous procedure, the UAV assumes that it is surrounded horizontally by other UAVs and enters the xy-blocked state. In this case, a reference not to move the UAV horizontally is sent, i.e., $\left|\right|v_{xy}^{ref}\left|\right|=0$. UAVs around are expected to leave at some point toward their destinations after circumventing the blocked UAV.

The states described above govern the horizontal UAV movement but do not affect its vertical movement. This allows UAVs to fly up or down toward their goal as they perform horizontal avoidance maneuvers, which is more efficient. However, as they change altitude they may find z-conflicts, blocking their vertical movement.

**z-blocked**: In this state the UAV has detected a z-conflict that precludes it from keeping its vertical movement. Otherwise, the UAV may appear unexpectedly in a horizontal plane where there are other UAVs without time to brake. In this state, the altitude of the UAV is blocked ($v_{z}^{ref}=0$), so there is no vertical movement.

**z-free**: This is the normal operation mode for the vertical movement. If no z-conflicts are detected, or the existing ones do not interfere with the path to the goal (e.g., a z-conflict above but the UAV is going down), the UAV can move up or down toward its goal at a speed proportional to the distance and bounded by the maximum allowed speed $v_{max}$ (Line 23, Algorithm 1).

## 5. Discussion

In this section, we discuss several aspects of our algorithm 3D-SWAP in more detail. In summary, 3D-SWAP is thought as a reactive algorithm for collision avoidance, so its primary objective is safety. Under certain realistic conditions, the algorithm can converge to a solution in most cases, even though not the optimal one. Besides, 3D-SWAP achieves robustness by fusing information from communication channels and onboard sensors, as well as modeling uncertainty in sensor measurements. Given its decentralized fashion, 3D-SWAP relies on local observations and communication, being scalable with the number of UAVs. Next subsections provide further discussion about these aspects.

### 5.1. Convergence

The ultimate goal of 3D-SWAP is to drive all UAVs to their destinations safely, i.e., without collisions. Under our assumption of detecting all static obstacles and neighboring UAVs in time, the safety can be guaranteed, since vehicles have always enough distance to brake and avoid collisions. For that, we need to bound the braking distance for the vehicles, the uncertainty of the sensors and have enough detection and communication ranges, which is realistic. Moreover, given certain conditions, the algorithm converges to a solution where all conflicts are solved eventually.

First, all obstacles must be cooperative teammates or static obstacles. With other kind of dynamic obstacles, our UAVs could get stuck since others would not follow 3D-SWAP rules. Besides, the braking distance for those external dynamic obstacles is not modeled, so collision-free maneuvers are not ensured in that case.

Second, the goals for all UAVs need to follow configurations where deadlocks are avoided. For instance, if several UAVs reach their goals and they surround the goal of another one with no space to get there without conflicts, the last UAV would never reach its destination. Another deadlock may occur when UAVs block their altitudes due to a z-conflict. Imagine that UAV 1 is on top of UAV 2, with $g_{1}$ below UAV 2 and $g_{2}$ on top of UAV 1, all aligned in the same vertical axis. They both would navigate to their destinations until a z-conflict occur, and they would get stuck pushing each other endlessly. Also, there may be deadlock situations when the goals for some UAVs are located in areas occupied by other blocked UAVs. The ones surrounding the blocked ones would stay circling around infinitely.

These deadlock situations are marginal and unlikely, but they could be solved by a higher-level motion planner that gets UAVs out of their deadlocks. Note that 3D-SWAP does not pretend to be a complete motion planning algorithm, but a reactive algorithm for collision avoidance. Our previous work [4] analyzes further deadlock situations for 2D scenarios. Nonetheless, they are unlikely if we do not consider crowded, confined scenarios. Therefore, a similar analysis of 3D-SWAP for a 3D scenario is out of the scope of this paper.

### 5.2. Optimality and Robustness

Even though 3D-SWAP can solve all conflicts under certain assumptions and keep UAVs safe without colliding, the solution is not optimal. UAVs could get blocked by others during some time or they may perform longer detours in highly conflictive situations. Note that we enforce counter-clockwise roundabouts, even if moving in the other direction were more efficient.

However, 3D-SWAP behaves in a robust manner in several aspects, even under unreliable communication and noisy sensors. First, the system is redundant fusing information from the communication channel and the onboard sensors. On the one hand, if communication fails the algorithm can still work, since the onboard sensors can be used to detect UAVs around. Note that in that case those obstacle points would be treated by default as static in the COD instead of dynamic. However, if communication failures were detected, we could treat all obstacles as dynamic to be conservative. Also, if neighbors positions were tracked, we could determine which angle bins should by dynamic, even with short communication losses. On the other hand, onboard sensors can have limited field of view due to processing or payload issues. This is why we also integrate measurements coming from communication channels. Moreover, the onboard sensor will usually point forward if its field of view is limited. Although 3D-SWAP assumes holonomic vehicles that do not need to turn around in order to navigate in any direction, the yaw of UAVs can also be controlled so that they always points their nose toward their forward direction. In that case, it is unlikely that an obstacle provoking a relevant conflict is not detected.

Besides, 3D-SWAP relies on the fact that UAVs will not get too close when avoiding each other, as they try not to overlap their reserved cylinders. However, even if these cylinders are appropriately designed, some unexpected behaviors may still arise due to external perturbations like strong wind gusts, saturated controllers, etc. In order to address those and make the system more robust, 3D-SWAP includes an additional controller to keep distances between UAVs above a threshold. This controller implements a repulsive force on the UAV when an external obstacle invades its reserved cylinder more than $d_{br}/2$.

Finally, 3D-SWAP is robust in terms of synchronization. UAVs do not need to execute the algorithm at the same time synchronously. Instead, each UAV resolves its conflicts locally and start its avoidance maneuvers asynchronously. Still, 3D-SWAP can guarantee safety as UAVs navigate, converging to the solution in a distributed fashion. As it will be explained in the experimental section, we use time synchronization for our UAVs, but this is just to ease logging tasks.

### 5.3. Scalability

3D-SWAP is a decentralized algorithm and it does not require high computational overhead, since the UAVs do not plan ahead but operate reactively. Each UAV runs locally Algorithm 1 only with local information from sensors and neighbors, so the approach scales independently of the number of UAVs. The main computation lies behind updating and analyzing the COD. The cost of these operations depends mainly on two factors: the size *M* of the pointclouds provided by the onboard sensors; and the level of discretization $N_{\varphi }$ in the COD. As *M* or $N_{\varphi }$ increase, the number of times that the COD needs to be updated or the number of angle bins to search for conflicts grow, respectively. In both cases, the increase of complexity is linear in the worst case and can be bounded by selecting the parameters *M* and $N_{\varphi }$. In practice, *M* is most critical for the complexity of the algorithm, since pointclouds are usually large for realistic fields of view.

## 6. System Integration and Experiments

In this section we show how 3D-SWAP was implemented for a team of UAVs. We describe our aerial platforms, the process followed for system integration and the experimental results to validate 3D-SWAP. We present results simulating the system with a Software-In-The-Loop approach, in order to showcase typical behaviors of the algorithm and evaluate its performance. Then, we detail our field experiments with up to 3 UAVs in an outdoor scenario.

### 6.1. Aerial Platforms

**Figure 4:**
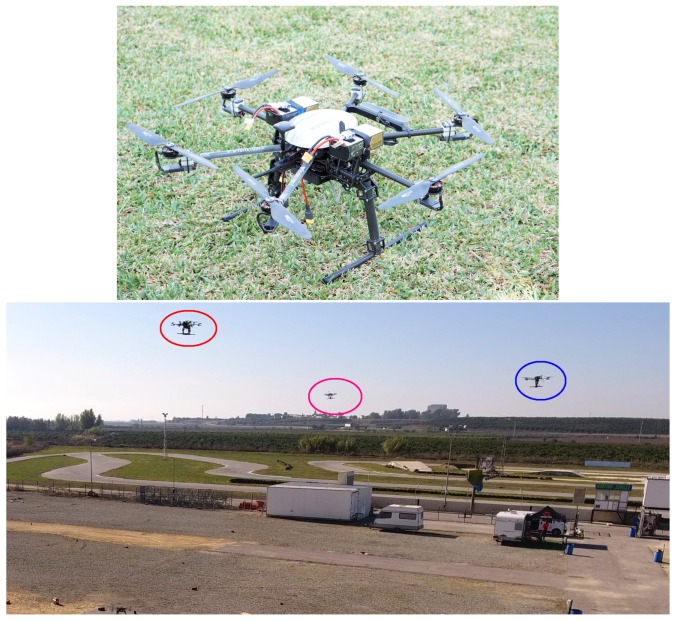
On **top**, close view of one of our custom-designed UAVs. At the **bottom**, a view of the experimental site. The colored circles indicate three flying UAVs.

The UAVs that we used in our experiments are the custom-designed hexarotors shown in Figure 4. They are made of carbon fiber and have a size of 1.18 × 1.18 × 0.5 m (including rotor blades). Their weight is 5.5 kg (including batteries, sensors and electronics), with a maximum take-off weight of 10 kg and a flight time of 20 min. Each UAV is equipped with: an Ubiquiti Rocket M5 5.8 GHz radiolink for communication with a ground station and other UAVs; a Lidar sensor based on a ZED stereo camera that provides 3D pointclouds for navigation; a 3DR uBlox GPS receiver for localization; a Pixhawk autopilot for UAV control; and an Intel NUC i7 processor (16 GB RAM).

### 6.2. System Integration

**Figure 5:**
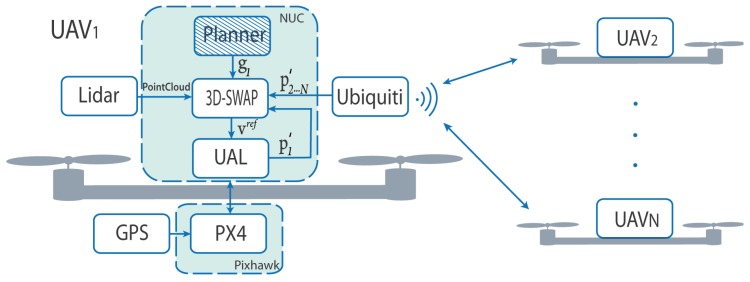
Block diagram of the system integration. Each UAV carries onboard a Pixhawk autopilot with GPS and an Intel NUC to run the navigation software. External obstacles are detected by a Lidar and the Ubiquiti is used as communication channel to share positions with others. The Planner is optional and would provide goals at each moment for the UAV.

We implemented 3D-SWAP with ROS Kinetic Kame (the code can be found at https://github.com/multirobot-ferr/3d-SWAP). A diagram block of the system integration can be seen in Figure 5. In particular, each UAV executes on its Intel NUC onboard an instance of a ROS node running 3D-SWAP (it runs at 10 Hz in all our experiments). This module could receive the goal of the UAV from a higher-level Planner, we just implemented a simple version for our experiments. We use the Lidar sensor onboard to provide poinclouds from external obstacles around and the communication channel through the Ubiquiti to share UAVs’ positions. This information is also collected in our ground station, and used for data logging and visualization.

Our 3D-SWAP is built on top of a UAV Abstraction Layer (UAL) (code at https://github.com/grvcTeam/grvc-ual), which is a software developed by our lab to simplify the interaction of external modules with the UAVs regardless of the autopilot used underneath. Our UAL offers interfaces to issue commands such as *take off*, *land* and *go to waypoint*, as well as reference velocities to command the autopilot. Our UAVs use the autopilot software PX4, which is abstracted by the UAL. Since we do not have an accurate RTK-GPS localization, we use the local coordinates provided by the PX4 filter (fusing GPS measurements) instead of its global coordinates, which are less stable. Measuring the initial UAV positions, the local coordinates can be transformed into a global coordinate system to be shared between the teammates. Moreover, for time synchronization between the nodes run on each UAV, we use Network Time Protocol (NTP) with a server on the ground station. This time synchronization is just for logging tasks, UAVs do not need it to run 3D-SWAP.

In order to ease system integration, we also developed a simulator based on Gazebo following a Software-In-The-Loop (SITL) scheme. The actual software of the PX4 is integrated within the simulator [23] to achieve realistic simulations where the system does not distinguish between real or simulated flights.

### 6.3. Simulations

We simulated 3D-SWAP with our SITL tool to evaluate its performance. The objective is to assess the robustness of 3D-SWAP under noisy sensors or how sensitive is to variations on its parameters. We used a 3D simulated scenario without external obstacles and simulated our aerial platforms in Section 6.1. In this scenario, 4 UAVs are placed in opposite squares of a 20 × 20 × 20 m cube, two UAVs start at the top part and the others at the bottom. The UAVs are commanded to exchange their positions. This creates a remarkable conflictive situation in the middle of the cube, where all UAVs arrive at the same time.

3D-SWAP parameters were set as: $r_{c}$ = 0.85 m, $h_{c}=h_{r}$ = 7 m, $r_{r}=r_{b}$ = 2.35 m, $h_{b}$ = 12 m, $N_{\varphi }$ = 360. The maximal speed of the UAVs during the simulation was $v_{max}=v_{a}$ = 2.5 m/s. We selected these sizes for the reserved and blocking cylinders after checking that the behavior was adequate in that scenario. Note that there is no wind in the simulator and dynamics are softer than with the actual platforms, so braking distances are shorter. Therefore, we can get UAVs closer in simulation than in field experiments (see Section 6.4) and test 3D-SWAP behavior in that situation. Since there are no external obstacles in the scenario and UAVs can communicate their positions, the Lidar is not strictly needed and was not used unless specified. In any case, we set the communication ($r_{comm}$) and detection ranges ($r_{det}$) large enough to detect all conflictive obstacles.

We performed a first simulation to evaluate the robustness of 3D-SWAP against noisy measurements. For that, we kept fixed the sizes of the reserved and blocking cylinders and increased gradually the noise in the UAVs’ measurements. In particular, we added a Gaussian noise on each coordinate of zero mean to the positions shared by the UAVs through the communication channel. We repeated the simulation (using always the same starting and goal positions for UAVs) with different values of the standard deviation of the added noise σ = {0 m, 1 m, 1.5 m}, 15 runs for each value. Since we have 4 UAVs in symmetrical conditions, we can extract 60 samples for the evaluation metrics in each case. All simulations were run on a single computer with the SITL simulation and the 3D-SWAP modules for the 4 UAVs. The computer had an Intel(R) Core(TM) i7-7700@3.60GHz CPU with 16 Gb RAM.

We used three metrics to evaluate the performance of 3D-SWAP. The clearance distance is the distance on the horizontal plane of a UAV to its closest obstacle, i.e., another UAV or external obstacle. It indicates the risk of collision on the xy plane. The traveled distance is the distance that a UAV covered to reach its goal location; whereas the traveled time is the time that took it to get there. These two last metrics assess how efficient the navigation is. We can compare the values with the nominal ones, i.e., the distance and time that a UAV would take to reach its goal without obstacles. We compute these nominal values assuming that the UAV would navigate in a straight line between the starting and goal positions at its maximum speed.

**Figure 6:**
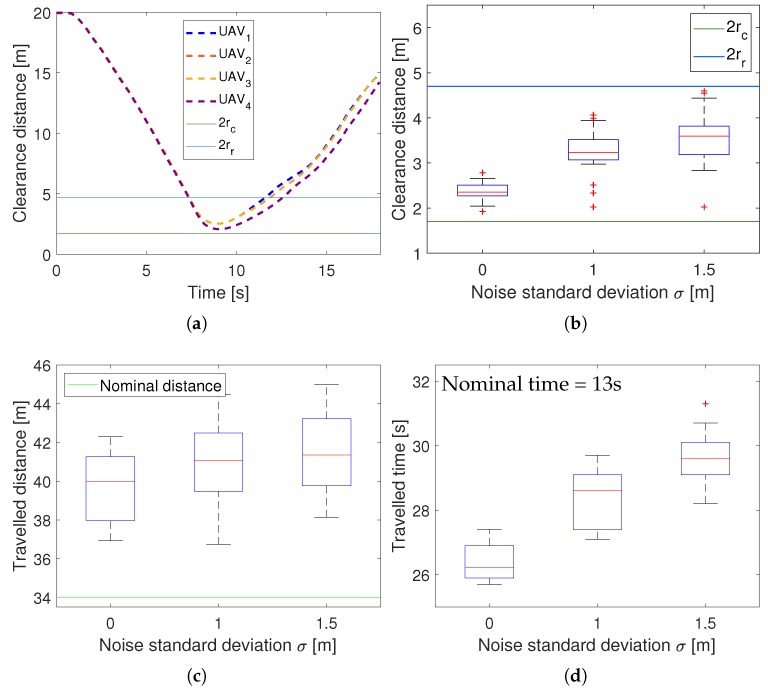
3D-SWAP results in simulations increasing noise in observed positions. (**a**) Clearance distance along time for a simulation run without noise. The distance $2r_{r}$ indicates when UAVs enter in xy-conflict, and the distance $2r_{c}$ when they collide. (**b**) Boxplot comparing clearance distances as noise increases. (**c**) Boxplot comparing traveled distances as noise increases. (**d**) Boxplot comparing traveled times as noise increases.

Figure 6 depicts the results of the 3D-SWAP performance for the simulations increasing the level of noise in the observed positions. Figure 6a shows an example of the evolution of the clearance distance during a simulation run. Similar runs gave similar results. In the middle, UAVs create a virtual roundabout to resolve the conflictive situations, performing an avoidance maneuver between second 7 and 13, approximately. It can be seen that they never collide. In Figure 6b, it is shown that the clearance distance does not decrease with noise, but slightly the opposite indeed. This depicts how 3D-SWAP is robust to noise in terms of safety, since UAVs do not get closer when their sensors are noisier. Due to the noise in the positions, UAVs think that others are closer than they really are, and trigger their maneuvers to solve conflicts even before. The distributions of the traveled distance and the traveled time are shown in Figure 6c,d. 3D-SWAP is not optimal and, in order to solve this conflictive situation, the traveled distance increases on average a 14% over the nominal value, whereas the traveled time increases a 50% over the nominal time. In terms of traveled distance, the solution is quite efficient indeed, not so in terms of the time spent in the roundabout. However, it is important to remark that the increase in the level of noise does not provoke a significant degradation in both metrics.

We performed a second experiment to test the effect of varying the size of the reserved cylinder, which is a key parameter in 3D-SWAP. Thus, we set a high and fixed level of noise σ = 1.5 m while we increased the reserved radius gradually $r_{r}$ = {2.3 m, 3.3 m, 4.3 m}. Again, we run 15 simulations for each value, obtaining 60 samples for each metric.

**Figure 7:**
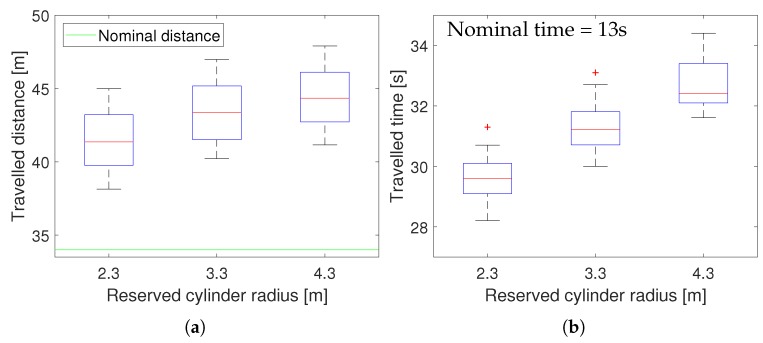
3D-SWAP results in simulations increasing reserved radius. (**a**) Traveled distance. (**b**) Traveled time.

Figure 7 shows the results for this experiment in terms of traveled distance and traveled time. It can be seen that they both increase linearly as the reserved radius does. This increase is not quite pronounced and is an expected behavior, since UAVs detect conflicts before and perform avoidance maneuvers longer.

**Figure 8:**
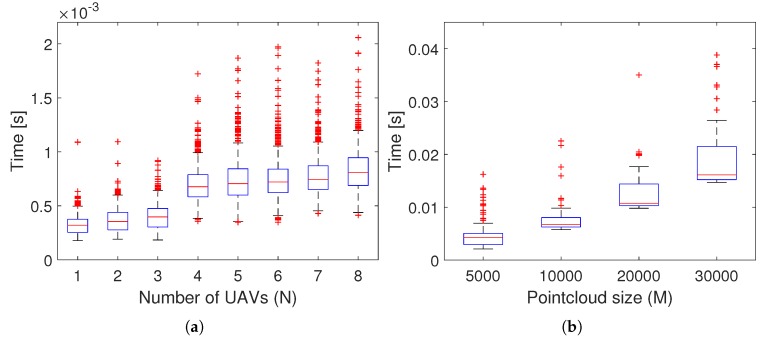
Scalability tests for 3D-SWAP. (**a**) 3D-SWAP execution time as the number of UAVs increases. (**b**) 3D-SWAP execution time as the pointcloud size increases.

Finally, we performed some scalability tests to analyze the computational overload of 3D-SWAP. First, we run the same cube simulation increasing the number of UAVs and computed the average execution time of 3D-SWAP (200 samples for each number of UAVs). Figure 8a shows how the execution time stays almost constant. Indeed, it should not increase with the number of UAVs given the decentralized nature of 3D-SWAP. However, the slight increment is due to the fact that the complete simulation was run on a single computer instead of distributively. Then, we run a simulation with a single UAV using the Lidar with different pointcloud sizes and measure the execution time (200 samples for each pointcloud size). Figure 8b shows how the execution time increases with the pointcloud size, since there are more points to process. As it was explained in Section 5, this is a critical parameter for the computational load of the algorithm that need to be selected according to onboard capabilities.

### 6.4. Field Experiments

We evaluated 3D-SWAP during several days of field experiments in an outdoor experimental site of 130 × 80 m that is located close to Seville (see Figure 4). We flew up to 3 UAVs following a strategy with several incremental phases.

#### 6.4.1. Tuning Parameters

We performed some simple experiments to calibrate the system and determine some parameters of the UAVs. In these field experiments there were no external obstacles, so we used the wireless communication channel (Ubiquiti) to exchange UAV positions and deactivated the Lidar sensors. The communication range $r_{comm}$ was enough to detect all conflictive UAVs in the scenario.

First, we need to know the uncertainty involved in the positioning systems of the UAVs to tune 3D-SWAP. We do not use RTK GPS and we have no ground truth either, so we could only get an empirical estimation of the localization error. For that, we took large sets of measurements of a UAV at different static positions and compared them with the average value to measure maximum errors of $\epsilon_{xy}$ = 1.5 m and $\epsilon_{z}$ = 0.1 m. Note that the altitude was provided by a highly accurate laser altimeter, and hence the much lower vertical uncertainty.

Second, to measure the braking distances, we left one UAV hovering while a second one was commanded to drive towards the first. We set up the reserved cylinders for 3D-SWAP large enough in altitude, in order to perform those tests with the UAVs at different altitudes but still provoke the braking. We found that under average windy conditions our UAVs were not able to stop in less than $d_{br}$ = 2.25 m on the horizontal plane, and $z_{br}$ = 2 m for the altitude. This allowed us to set $r_{r}=r_{b}$ = 4.6 m and $h_{b}$ = 12 m. We used different parameter values depending on the windy conditions, but we report here those for the experiments included in the paper. $r_{c}$ = 0.85 m and $h_{c}=h_{r}$ = 7 m were given by the size of the UAVs and the downwash effect (a minimum separation in altitude is required to avoid perturbations). Finally, we limited for safety the maximal speed of the UAVs to $v_{max}=v_{a}$ = 1.5 m/s in all our field experiments and set $N_{\varphi }$ = 360.

#### 6.4.2. Results

Once the parameters were tuned, we started testing 3D-SWAP with preliminary tests where the UAVs flew and exchanged positions at different horizontal planes. In particular, we set $h_{r}$ to quite large values in order to enforce xy-conflicts even with UAVs at different altitudes (i.e., infinite reserved cylinders). Then, we run several tests with two UAVs at antipodal positions of a circle of 10 m radius, exchanging their positions. Finally, both UAVs were commanded to the center of the circle to check that 3D-SWAP was ensuring safety in case of overlapping destinations. We experienced that the UAVs were quite sensitive to the level of wind, being necessary to adjust the sizes of the cylinders depending on those conditions.

**Figure 9:**
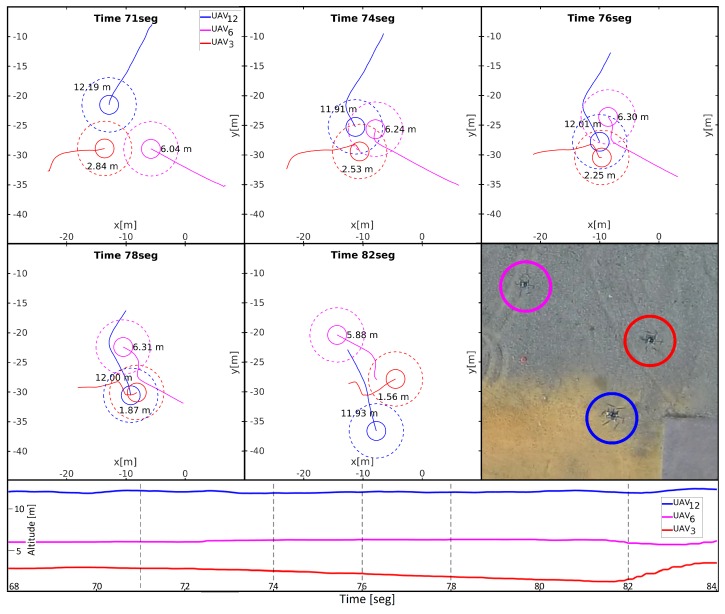
Different snapshots with the paths followed by the 3 UAVs during a field experiment. The last snapshot is the final view of the actual experiment. Solid circles represent collision cylinders and dashed circles reserved cylinders. Each UAV is labeled with its altitude. At the bottom, the evolution of the altitudes of the UAVs along the experiment are shown, together with marks on the time instants when the above snapshots were taken.

After the first preliminary tests, we run several experiments with 2 and 3 UAVs exchanging their positions at different altitudes without constraining their movement to different planes (without infinite cylinders). One of those experiments is depicted in Figure 9 (the video of such experiment can be found at https://youtu.be/4Eofi38RGWk). Three UAVs were placed in the vertices of a triangle and were commanded to navigate to their diametrically opposite position, creating a conflictive area in the middle. For each UAV the altitude of its starting and goal positions were equal and different to the other UAVs, flying each at 3, 6 and 12 m (from here on: UAV_3_, UAV_6_ and UAV_12_). Since UAVs do not need to vary their altitude to reach their goals, z-conflicts are not relevant in this experiment. If we center our attention in UAV_3_, it is possible to see how between seconds 71 and 78 it detects an xy-conflict with UAV_6_ and performs an avoidance maneuver on a horizontal plane to surround it counter-clockwise (rendezvous state). However, UAV_3_ detects no xy-conflict with UAV_12_, which is far enough in altitude, and hence they ignore each other crossing their horizontal trajectories. Instead, UAV_6_ is involved in a more complex situation, having xy-conflicts with the two other UAVs. In second 74 is possible to see how the UAV maneuvered to avoid UAV_3_ and starts a new maneuver to avoid UAV_12_ counter-clockwise too. From second 78 on, UAV_6_ is back to xy-free state and it navigates straight to its goal. Finally, UAV_12_ performs a slight change of direction from second 74 (rendezvous state) to head properly the virtual roundabout created with UAV_6_. After second 78, it is back to xy-free state and it flies toward its goal over UAV_3_ ignoring it. Note that UAV_3_ decreases its altitude when UAV_12_ flies over it due to the downwash effect.

## 7. Conclusions

We presented 3D-SWAP, a decentralized algorithm for multi-UAV collision avoidance in 3D outdoor scenarios. The primary objective of 3D-SWAP is to navigate the UAVs safely in a robust manner, even under harsh constraints. We do not assume accurate positioning systems outdoors, and our results show how 3D-SWAP ensures safety under noisy UAV positions. We propose a decentralized approach because it tackles better communication and computational issues. The algorithm integrates measurements from onboard Lidar sensors with positions communicated by other teammates, which provides additional flexibility. Moreover, 3D-SWAP behaves reactively instead of planning ahead in time, which is also relevant to achieve a low computational burden. Therefore, the algorithm is not optimal, but we proved it still efficient in terms of traveled distance. Our scalability tests also showed that 3D-SWAP scales with the number of UAVs and that its main computational load comes from the size of the Lidar pointcloud.

We used an SITL tool to integrate our algorithm and evaluate its performance 3D scenarios with highly conflictive situations. Furthermore, we run field experiments with inaccurate GPS localization. We found that external conditions such as localization errors or wind speeds are critical for the performance of the system (e.g., to determine UAV braking distances); and hence, 3D-SWAP parameters need to be properly adjusted beforehand. As future work we plan to run field experiments using Lidar sensors to detect other UAVs instead of the communication channels. We also plan to explore better the theoretical guarantees of our algorithm in terms of completeness, studying deadlock situations.
